# A Mixed Methods Study of Change Processes Enabling Effective Transition to Team-Based Care

**DOI:** 10.1177/1077558719881854

**Published:** 2019-10-15

**Authors:** Michael Anne Kyle, Emma-Louise Aveling, Sara Singer

**Affiliations:** 1 Harvard Business School, Boston, MA, USA; 2 Harvard T.H. Chan School of Public Health, Boston, MA, USA; 3 Stanford University School of Medicine and Graduate School of Business, Stanford, CA, USA

**Keywords:** team-based care, primary care, transformation, change

## Abstract

Team-based care is considered central to achieving value in primary care, yet results of large-scale primary care transformation initiatives have been mixed. We explore how underlying change processes influence the effectiveness of transition to team-based care. We studied 12 academically affiliated primary care practices participating in a learning collaborative, using longitudinal staff survey data to measure progress toward team-based care and qualitative interviews with practice staff to understand practice transformation. Transformation efforts focused on team formation and capacity building for quality improvement. Using thematic analysis, we explored types of change processes undertaken and the relationship between change processes and effective team-based care. We identified three prototypical approaches to change: pursuing functional and cultural change processes, functional only, and cultural only. Practice sites prioritizing both change processes formed the most effective teams: simultaneous functional and cultural change spurred a mutually reinforcing virtuous cycle. We describe implications for research, practice, and policy.

## Introduction

Multidisciplinary care teams are viewed as the basic operating unit of a learning health care system capable of generating ongoing improvements in quality and efficiency (American Academy of Family Physicians, American Academy of Pediatrics, American College of Physicians, & American Osteopathic Association [AAFP], 2007; Bielaszka-DuVernay, 2011; Schottenfeld et al., 2016). Thus, team-based care is a cornerstone of most primary care practice transformation initiatives and a requirement for credentials like the Patient-Centered Medical Homes (PCMH; AAFP, 2007; Bloniarz & Smalley, 2014; Bodenheimer, 2007; Grumbach & Bodenheimer, 2004; Iglehart, 2008; Mitchell et al., 2012; Naylor et al., 2010; Wagner, Coleman, Reid, Phillips, & Sugarman, 2012). Despite widespread optimism about the transformative potential of team-based care, in practice medical home models have delivered mixed results (Sinaiko et al., 2017). This article draws on a mixed methods, multisite study of primary care transformation to identify and characterize change processes that facilitate transition to effective team-based primary care. An outcome evaluation of the intervention studied here suggest the transformation was associated with lower utilization and costs among chronically ill patients (Meyers et al., 2019).

Theoretical and empirical literature suggests that effective primary care teams can improve outcomes of great interest, such as care coordination, quality, and efficiency (Alexander et al., 2005; Reiss-Brennan et al., 2016). Team-based care has been associated with better continuity, access, and satisfaction for patients (Grumbach & Bodenheimer, 2004), while team structures can enhance capacity for shared learning and improvement (Provost, Lanham, Leykum, McDaniel, & Pugh, 2015; Valentine & Edmondson, 2015). Supportive team structures and team-oriented culture may protect against burnout among primary care clinicians and staff, and improve satisfaction and motivation for clinical work (Brooks, Singer, Rosenthal, Chien, & Peters, 2017; Sheridan et al., 2016; Willard-Grace et al., 2014). Among physicians and physician trainees in particular, better relationships with practice personnel have been associated with improved job and career satisfaction (Brooks et al., 2017; Williams et al., 1999).

The potential of teams to facilitate systemic continuous improvement is central to the rationale for promoting team-based care. Existing research indicates that effective teams can be significant drivers of innovations that enable quality improvements (QIs) and efficiency gains (Lemieux-Charles & McGuire, 2006; Mitchell et al., 2012). In the context of primary care practice transformation, continuous progress toward greater quality and cost effectiveness requires more than equipping individuals with QI skills and assigning improvement work to individual staff (who may lack the support of the wider team or system); rather, it requires collective capacity and commitment at the level of the teams responsible for delivering care.

Despite the potential of team-based care, positive practice transformation results have not been achieved across entire populations or been reproduced consistently or at scale. Primary care reform efforts have yet to deliver on their promise of higher quality at lower cost to the dismay of clinicians, payers, and policy makers alike (Friedberg, Rosenthal, Werner, Volpp, & Schneider, 2015; Friedberg, Schneider, Rosenthal, Volpp, & Werner, 2014; Jaen et al., 2010; Levine, Linder, & Landon, 2016; McGlynn, Adams, & Kerr, 2016; Meyers et al., 2019; Peikes et al., 2018).

Models of team-based care recognize that implementation of effective teams is a complex undertaking with structural, procedural, and cultural dimensions (AAFP, 2007; Grumbach & Bodenheimer, 2004; Schottenfeld et al., 2016; Wagner et al., 2012). Yet team-based care is often assessed solely in terms of team structure, even as evidence suggests that team processes and team effectiveness are more strongly associated with priorities such as patient-centered care (Helfrich et al., 2014; Ovretveit & Gustafson, 2002). This pattern highlights the importance of characterizing implementation and change processes (not simply “inputs” and “outputs”) and identifying the change processes critical for success (Dixon-Woods, Bosk, Aveling, Goeschel, & Pronovost, 2011).

### New Contributions

This mixed methods, concurrent evaluation of primary care practice transformation identifies and characterizes change processes that support establishment of effective primary care teams and develops a taxonomy of approaches to change. We studied 12 academic primary care practices participating in a multiyear learning collaborative, which had as a central focus establishing team-based care and building capacity for continuous improvement (Bitton et al., 2014). The intervention design reflected current research on primary care transformation and team formation. Despite participating in the same improvement collaborative with the same “inputs,” participating practices varied considerably in their attainment of effective team-based care.

This research builds on the primary care transformation and team formation literature. We focus in particular on team formation processes, which have received less attention than other aspects of teaming (Helfrich et al., 2014; Ovretveit & Gustafson, 2002). We describe two central team formation change process types—cultural and functional—and show how the interaction between these change types is vital to establishing high-performing teams and effective transformation. This study thus provides empirical evidence of how variation in the way primary care practices undertake transformation affects the quality of the resulting team-based care. We develop a taxonomy of approaches to change that (a) can inform *how* practices should go about forming teams and (b) may offer a useful assessment or diagnostic tool for evaluating practices’ progress toward effective team-based care.

While we acknowledge that context significantly influences improvement intervention processes and outcomes (Bate, 2014; Ovretveit, 2011), our analysis focuses on characterizing within-practice transformation processes and their influence on the effectiveness of team-based care. Exploring contextual influences on practices’ capacity or propensity for adopting particular approaches to change represents an important area for future research.

### Conceptual Model

The primary care transformation intervention studied in this article yielded mixed results, despite drawing on evidence of best practices for creating primary care teams. Given substantial evidence about the features and structure of high-performing teams, we focused on characterizing and comparing the processes by which practices pursue these changes as a potential factor distinguishing the effectiveness of the resulting teams.

Our assessment of the practices’ transition to effective team-based care draws on existing theory of effective primary care teamwork. We define effective team-based primary care in terms of a team’s ability to deliver three widely endorsed objectives of team-based care (AAFP, 2007; Agency for Healthcare Research and Quality, 2016; Wagner et al., 2012): (a) patient-centered care, meaning services that foster interpersonal relationships and improved quality and efficiency; (b) capacity for continuous improvement; and (c) enhanced clinical work satisfaction, through sharing tasks and responsibilities across team members. These objectives broadly align with the three recognized objectives of teams more generally: achieving the team’s shared goal, improving as a team, and growth of individual members (Hackman, 2002).

There is an extensive evidence base describing factors that contribute to effective primary care teams (Lemieux-Charles & McGuire, 2006; Schottenfeld et al., 2016). Research focused on *implementing* team-based primary care suggests that actions that support effective teaming include participation in a learning collaborative, defined team structure and roles, regular team meetings, teamlet huddles, inclusive leadership, and data systems (plus training) to support QI (Giannitrapani et al., 2016; Grace, Rich, Chin, & Rodriguez, 2015; Helfrich et al., 2014; Helfrich et al., 2016; Rodriguez, Meredith, Hamilton, Yano, & Rubenstein, 2015). Yet even with evidence-based guidance on how to design and implement teams, primary care practices are not always able to achieve effective team-based care (Sinaiko et al., 2017).

While elements of effective team formation and performance have been articulated (Agency for Healthcare Research and Quality, 2016; Helfrich et al., 2014; Lemieux-Charles & McGuire, 2006), how these features interact with one another is less clear. Complexity theory may offer a useful, additional frame for understanding primary care team formation and performance (Stroebel et al., 2005). Viewing health care organizations as complex adaptive systems leads us to recognize that relationships among component systems are not always linear; they may interconnect and interact in unpredictable ways (Plsek & Greenhalgh, 2001). This suggests the need to consider how team activities, processes, and characteristics relate to each other and to performance outcomes (Davidoff, 2009). Drawing on complexity theory, we analyze how different types of primary care team change processes interact with one another to drive the effectiveness of the resulting teams.

## Method

We used mixed methods to explore the relationship between team formation change processes and quantitative measures of team performance. We triangulated quantitative measures of team performance, as reported by practice employees (clinicians and staff), with qualitative interviews exploring clinician and staff experiences of the transformation process (Wisdom, Cavaleri, Onwuegbuzie, & Green, 2012).

### Study Context

The Academic Innovations Collaborative (AIC) is a learning collaborative that was established to support practice transformation in a cohort of primary care teaching practices (Bitton et al., 2014). Like primary care and safety net medical home initiatives, the goal of the AIC was to build high-functioning teams, capacity for population health management, and patient engagement.

Initial AIC membership comprised 18 practices from six health systems. Practices applied to participate in the AIC and received both financial support (a grant) and technical support (QI coaching, monthly training webinars, site visits, and triannual learning sessions). In the first 2 years, the AIC focused primarily on transitioning to team-based care. Practices also worked to empanel patients, promote patient engagement, and build skills for continuous improvement, such as the ability to use Plan-Do-Study-Act (PDSA) methods. The third year of the collaborative focused on specific improvement initiatives: colorectal cancer screening for adult practices and early intervention for pediatric practices.

The teams we study in this analysis are the product of an intervention based on existing evidence of primary care team formation. The intervention established formal teams, explicitly recognizing the interdependence of primary care clinicians and staff in the context of practice transformation, and sought to facilitate change processes enabling teams to work effectively (Lemieux-Charles & McGuire, 2006; Song et al., 2015). To establish formal teams, the AIC asked practices to assign, in writing, all practice staff to an interdisciplinary group of people consisting of physician(s), medical assistant(s), and other staff, including nurses, social workers, administrative assistants. While part time schedules meant that there were some changes in team composition from day to day, team assignments aimed to create cohesion and continuity in work relationships. In some cases, patient panels were attributed to teams. Prior to establishing teams, practices worked in a dyadic model where a physician and medical assistant were paired together based on who was present during a given clinic session, and staff in other roles would interact with colleagues and patients as needed.

As part of the intervention, a learning collaborative provided technical assistance around key facilitators of transformation, including methods for QI, team-building and training around the collection and use of data (Stockdale et al., 2018). Both practice-level and organizational leaders were encouraged to champion team-based care and received protected time to commit to the transformation effort (Grace, Rich, Chin, & Rodriguez, 2014; Stockdale et al., 2018). Teams were urged to hold routine, structured meetings to work together on QI goals (Grace et al., 2014; Helfrich et al., 2016). Team members were advised to form “teamlets,” which were small units comprising the physician and immediate colleagues in a given clinic session, such as the medical assistant and/or registered nurse (Rodriguez et al., 2015). In-person teamlet huddles at the beginning of the day were promoted as a key teaming strategy (Gale et al., 2015; Rodriguez et al., 2015).

A concurrent, 4-year evaluation of the AIC showed variation across participating practice sites in terms of team dynamics, clinical work satisfaction, and patient care coordination (Brooks et al., 2017; Sheridan et al., 2016; Song et al., 2015). The mixed methods study reported here, undertaken at the end of the AIC’s third year, represents one component of the larger evaluation, which also included assessment of the impact of the intervention on health care quality and costs (Meyers et al., 2019). This research was approved by the Harvard T.H. Chan School of Public Health Office of Human Research Administration.

### Sample

Of the 18 practices participating in the AIC at the time of our study, we selected 12 using blinded scores from annual staff surveys assessing team dynamics and professional satisfaction (described further below). To enable exploration of factors that facilitated and impeded the transition to team-based care, we included six practices with high-mean team dynamics scores on Year 3 of the AIC staff surveys and six practices with low-mean team dynamics scores on the same survey. All 12 practice sites selected for this study agreed to participate. Though clustered within six health systems, practice sites were considerably diverse including very large (over 300 employees) to very small (under 50 employees) sizes, four hospital-based and eight community-based sites, and serving substantially different mixes of patients (Table 1). Ten of 12 practices were engaged in structured QI activities in addition to the AIC. In all practices, the AIC was the most significant QI intervention. Other (coexisting) QI initiatives involved targets (e.g., breast cancer screening referrals) but did not provide training or technical assistance to support practice changes associated with new organizational goals. One site was part of an institution that had a more comprehensive program to support QI, but this was targeted at individual skill-building, not practice-level change; individuals from the practice could elect to participate in leadership training related to a QI project they wanted to undertake.

**Table 1.** Sample and Practice Characteristics.

Site	Interviewees	Practice size		Practice site	Structured QI in addition to AIC
		Total staff	Patient visits per year		
1	6 (Medical Director, 2 MDs, AA, PM, NP)	<50	<10,000	Community hospital	No
2	3 (Medical Director, MD, RN)	<50	<10,000	Community hospital	Yes
3	2 (Medical Director, PM)	<50	10,000-20,000	Community practice	Yes
4	5 (Medical Director, MD, RN, MA, AA)	>150	>50,000	Academic Medical Center	Yes
5	4 (Medical Director, MD, LPN, LCSW)	>150	>50,000	Academic Medical Center	Yes
6	4 (Medical Director, MD, PM, CC)	>150	>50,000	Academic Medical Center	Yes
7	4 (Medical Director, MD, MA, AA)	<50	10,000-20,000	Community practice	No
8	5 (Medical Director, MD, PM, RN, LCSW)	>150	20,000-50,000	Academic Medical Center	Yes
9	3 (MD, RN, LCSW)	50-100	>50,000	Community practice	Yes
10	3 (MD, RN, LCSW, PA)	<50	20,000-50,000	Community practice	Yes
11	3 (Medical Director, RN, practice manager)	<50	20,000-50,000	Community practice	Yes
12	5 (Medical Director, 2 MDs, psychologist, RN)	50-100	20,000-50,000	Community practice	Yes

*Note*. QI = quality improvement; AIC = Academic Innovations Collaborative; AA = administrative assistant; RN = registered nurse; NP = nurse practitioner; LCSW = licensed clinical social worker; PA = physician assistant; PM = project manager; LPN = licensed practical nurse; MA = medical assistant; MD = medical doctor; CC = care coordinator.

### Data Collection

#### Survey Data

Data on team dynamics and clinical work satisfaction came from annual administration of the Primary Care Team Dynamics Survey and a rating of clinical work satisfaction (Song et al., 2015) to all patient-facing staff at each practice site. The 26-item survey covers five domains of teamwork, including questions about skill sets of teams, communication within teams, shared goals and understanding of each other’s roles, and perceptions of mutual respect and trust, plus one satisfaction measure. All items use a 5-point Likert-type scale, where 1 = *strongly disagree* and 5 = *strongly agree*. Survey response rates were 70%, 67%, 66%, and 57% in Years 1 through 4, respectively. The number of respondents ranged from 995 to 1,082 annually. In this study, we used this survey data in two ways: (a) using blinded data from the Year 3 survey, we selected the six highest scoring and six lowest scoring practices for participation and (2) we then combined unblinded survey results from all 4 years with qualitative data to explore how practices with high- and low-performing teams varied in their approach to change.

#### Interview Data

At each practice, we sought to interview the person designated as principal investigator for the AIC work (typically the Medical Director), the day-to-day leader overseeing the site’s participation in the AIC, and one each of a frontline physician, nurse, and person in a care coordination role. Where practices did not have staff filling specific roles (e.g., nurse), we omitted these interviews. Where practices had multiple people in a given role, the study team selected an interviewee randomly from the practice personnel list, or selected a staff member who was available on a day we proposed to conduct interviews. In all, we invited 52 individuals and completed 48 interviews; two invitees did not respond and two could not be interviewed due to turnover. We conducted three interviews by phone; the rest were completed in-person.

The 48 staff members interviewed included 22 physicians, 8 nurses, 7 staff in a care coordination role (e.g., social worker, care manager), 4 program managers (administrative staff dedicated to the AIC and/or similar innovation projects), 3 administrative assistants, 2 nonphysician day-to-day leaders (a physician assistant and a psychologist), 1 medical assistant, and 1 nurse practitioner. We interviewed an average of four staff members per site (range two to six). All interview subjects provided either written or recorded verbal informed consent.

One or two researchers from the independent evaluation team (MAK, ELA, and SS) conducted the interviews, which lasted approximately 45 minutes. The semistructured interview guide (available on request from the corresponding author) explored experiences of the transition to team-based care, including views on and experiences of changes at the practice and individual levels; reflections on improvement efforts, including specific initiatives and more generalized efforts to develop capacity for continuous improvement within the practice; perceptions of the factors that supported and inhibited establishing teams and making improvements; and more general reflections on changes in the practice over the 3 years of the AIC. All interviews were audio recorded and transcribed.

### Analysis

#### Characterizing Team Performance

Team dynamics scores reflected individual respondents’ ratings of their practice on constructs from the Team Dynamics Survey directly inquiring about team performance, as well as constructs characteristic of effective teams (e.g., skills necessary to perform assigned work, good communication, and professional satisfaction). We calculated a team dynamics score for each practice by averaging the score of all individuals within that practice. These were used to select the six highest and six lowest scoring practices into our sample, though study team members were initially blinded to practices’ high or low designation. After completing interviews and qualitative analysis, we unblinded all 4 years of survey data, looking in detail at practice-level scores. We designated practices as “high” or “low” performers based on patterns of team dynamics and clinical work satisfaction scores over time. High performers, achieving “effective team-based care,” were practices that had persistently high or consistently increasing scores on both team dynamics and clinical work satisfaction. There were two types of low performers: (a) practices that had persistently low or consistently declining team dynamics and satisfaction scores and (b) practices that had fluctuating scores, scoring highly in some years and low in others without a clear trend, as well as high variance in scoring across constructs.

#### Characterizing Change Processes Using Interview Data

Through interviews, we sought to understand the types of change processes employed and how these affected the ability of practices to attain effective team-based care. Analysis thus focused on (a) identifying and characterizing change processes underpinning the transformation to team-based care and (b) exploring the interrelationships between types of change process and attainment of effective team-based care.

To identify change processes and explore their impact on team effectiveness, we used a thematic network method of analysis (Attride-Stirling, 2011), supported by NVivo 10 software. Thematic network analysis proceeds by first identifying basic themes, then grouping these into high-order themes, and analytically exploring the relationships among themes. We first developed a codebook of a priori themes derived from our research questions and informed by the literature (e.g., perceptions of patient-centered care, the role of the AIC). Having applied these to the data, we then continued coding to elaborate a set of basic themes. To these deductively derived themes, we added further, descriptive basic themes that emerged in the process of coding transcripts (e.g., role revision, openness to experimentation). During the analysis phase, the research team met weekly or biweekly to review and discuss basic themes and clustered them into organizing themes (see Supplemental Appendix A, available online, for a priori and emergent themes). This interpretive work focused on consolidating, clarifying, and grouping basic themes into higher order themes. Our analysis ultimately differentiated two aspects of the transformation to effective team-based care: team formation and building continuous improvement capacity. Basic themes associated with each of these domains clustered into two categories of change process: functional and cultural. It was through the process of visually mapping themes and their relationships to one another and to larger themes of function and culture that we recognized the centrality of interdependence and recursivity. That is, we recognized the ties between function and culture as we repeatedly struggled to assign basic codes to one or the other. For example, “data” seemed at first an obvious match to function, yet culture was powerfully reflected in quotes about data.

We cycled through these steps multiple times, iteratively refining and revising basic and organizing themes. The first author coded all data using the final combination of inductively and deductively derived basic themes. Within sites, we explored the relationships between functional and cultural change processes within and across domains to characterize each practice’s transformation approach. Last, we explored change patterns across practices.

## Results

### Variation in Team Performance

Despite substantial commonalities among participating practices (e.g., commitment and exposure to the same intervention, academic affiliation), quantitative and qualitative data indicated substantial variation in the quality and nature of team formation and continuous improvement capacity across sites. Survey data showed variation in team performance (see Supplemental Appendix B, available online) across constructs including communication, collaboration, and professional satisfaction. For example, the practice-average response to the prompt “our team is effective” ranged from 3.45 to 4.32 (mean 3.82). Average practice nonphysician clinical work satisfaction ranged from 3.33 to 4.56 (mean 3.80) in Year 4. The range for clinical work satisfaction among physicians (by practice) was broader: from 2.83 to 4.71 (mean 3.86).

We did not see an association of practice characteristics, like size and patient population, with team formation and improvement capacity. While we observed some similarities among practices within the same parent health system, practice sites that formed effective teams and built capacity for improvement were dispersed across large hospital-based clinics with a high-teaching burden and smaller community-based practices. Ten of 12 practices were involved in structured QI efforts in addition to the AIC; of the two practices without other structured QI, one was high-performing and one low-performing according to the team dynamics survey. All practices reported that the AIC was the focal transformation intervention; other structured QI initiatives were typically attached to performance measures without support for transformation. Practices uniformly described exporting lessons learned from the AIC to other QI efforts.

Through qualitative analysis, cultural and functional change processes emerged as the key drivers of practices’ success in transitioning to effective team-based care. Practices that engaged in both cultural and functional change were most successful at building effective teams, as assessed by team dynamics scores. These sites often found that taking on both dimensions of change at once was overwhelming at first, and felt that progress was slow. But, while it was messy and frustrating at the beginning, these practices gained momentum as they began to experience a virtuous cycle wherein cultural and functional change became mutually reinforcing. Committing the time and space to work through early conflict created the foundation for a successful transition to effective teams. On the other hand, practice sites with asymmetric change processes (prioritizing either culture or function) struggled to achieve effective teaming consistently or completely. With a narrower scope of change, these practices often got off to a quicker start, but progress became difficult without the momentum generated by the mutually reinforcing interaction between functional and cultural change processes. Practices prioritizing culture over function had good intentions and poor follow through, which may explain the year-to-year fluctuation in their team dynamics scores and staff satisfaction. Practices prioritizing function over culture struggled to create an environment conducive to change, and saw consistently poor or declining team dynamics and staff satisfaction.

We differentiated two “domains” (or aspects) of change in the process of establishing team-based care: (a) forming teams as an operational unit and (b) building team capacity for continuous improvement. The cultural and functional change processes characteristic of practices’ approach to transformation for each domain are described and summarized in Table 2.

**Table 2.** Functional and Cultural Change Processes Characteristic of Practices’ Approach to Transformation, by Domain of Change.

Domain of change	Functional change processes	Cultural change processes
Team formation	Role revision: Formalized reallocation of tasks and responsibilities. Team time: Shared time (huddles) and/or space (team sits together) to work as a team. Access to clinical data: Electronic records give all team members access to patient records and ability to take action.	Sharing authority: Devolve and share power, dialogue and two-way feedback. Staff engagement: Communicate transformation plans and encourage staff participation from the start. Physician leadership: As highest status personnel, unique role in modeling egalitarian behavior and in tackling resistant peers.
Capacity for continuous improvement	Improvement skills: Training, for example, Plan-Do-Study-Act, which enable staff to undertake improvement activities. Meeting structures: Systematic communication from teams to practice leaders and vice versa. Data collection capacity: Mechanisms for tracking progress toward performance goals, for example, clinical registries.	Openness to experimentation: People seek out opportunities to test ideas, comfort with a state of continual change. Willingness to fail: View failures as learning experiences rather than threats. Data as a valued tool: Feedback is sought out and viewed as a tool of empowerment rather than a mechanism for punishment.

### Team Formation

Central to team formation was establishing how to assign and accomplish work across practice staff. While team formation entailed some new tasks (e.g., for population health management), it mainly involved reconfiguring existing work (e.g., reallocating depression screening to a different team member). Functional change processes were those related to practical, operational aspects of teaming, specifically: role revision, team time, and shared access to clinical data. While functional changes that formalized role revision and established supportive structures were important, forming teams also required cultural changes, that is, change processes that acted on the normative and relational aspects of teaming. Successful role revision required sharing authority and flattening traditional hierarchies. In all practices, this was a difficult and often bumpy process, wherein both high- and low-status team members experienced challenges in delegating and assuming authority. For team time (e.g., huddles, team meetings) to be used effectively—or at all— staff had to be engaged in the transformation process and encouraged to participate. Physician leadership was essential to supporting team formation; at the team level, physicians had to relinquish tasks and authority to teammates, so their willingness to change shaped team function and culture. At the practice level, physicians in leadership roles had the unique authority to tackle active resistors among their colleagues (Saint et al., 2009).

### Building Team Capacity for Continuous Improvement

One of the goals of team-based care and practice transformation is improving the quality of care. Capacity building for continuous improvement built on efforts to develop effective team-based care. Whereas team formation concentrated on reallocating existing work from individuals to teams, continuous improvement necessitated an array of new tasks in the clinical environment. Exceeding the capacity of any one individual, this work depended on a team-level endeavor. A specific QI goal for the AIC teams was to improve colorectal cancer screening. However, practice sites differed in the application of the formal training they received through the AIC to screening initiatives and in the space they created for building improvement capacity in their practices.

Three functional change processes distinguished capacity building for continuous QI: developing continuous QI skills, data collection, and establishing practice-wide meeting structures. Improving colorectal cancer screening rates meant collecting, recording, and tracking data previously not accessible or not monitored. Process improvement skills, such as the ability to conduct PDSA cycles, became necessary to enable responsiveness to information generated through the screening process. Continuous improvement requires a potentially stressful state of being in continual change; openness to experimentation and willingness to fail were two crucial cultural aspects of continuous improvement. Teams open to experimentation identified improvement opportunities and tested new ideas. Willingness to fail meant that teams interpreted failure as a lesson rather than a threat. Finally, viewing data as a tool for empowerment rather than a mechanism for punishment was an important cultural adaptation to support the collection and responsiveness to new data.

Practice leaders played a pivotal role in continuous improvement. Since new tasks often required different or more resources, practice leaders interfaced with the parent system to advocate for changes which enabled practice-level functional and cultural changes. Examples included gaining/negotiating access to certain types of data and securing protected time for practice staff (particularly physicians) to focus on transformation.

### Recursivity

While functional and cultural change processes were individually important, they were most effective when mobilized in tandem. The recursive relationship between functional and cultural changes processes was key to the effectiveness (or not) of team-based care. High-performing practices prioritized both functional and cultural change, and benefitted from the mutually reinforcing dynamic between those processes. Cultural changes created an environment conducive to functional changes, and functional changes furnished support systems for cultural changes. Without functional changes, practices struggled to sustain cultural changes, as it required a constant reinvestment of activation energy from practice staff. Without cultural changes, functional changes were imposed on a relational context unable to support them.

For example, when forming teams, practice sites reported greatest success when they delegated tasks and distributed authority among team members. As practice culture evolved to permit shared authority among team members, nonphysicians took on more of the functional responsibilities of patient care, and the recursive relationship between culture and function became a virtuous cycle. This task-shifting relieved some of the burden on physicians and felt rewarding to nonphysicians, contributing to a culture of mutual regard and respect, which in turn reinforced confidence in, and reduced discomfort with, the redistribution of tasks and responsibilities. Giving nonphysician team members greater responsibility for certain elements of patient care (e.g., routine screenings, between visit calls, introduction as “your” nurse) created opportunities for more staff to form meaningful relationships with patients. These bonds with patients were a source of sustenance to all members of the practice. In environments where people often felt overburdened and underresourced, patient relationships enabled staff to derive meaning and see the tangible impact of their work. At the same time, these new points of contact gave staff opportunities to glean insights or make contributions to patient care that might not otherwise happen. Practice staff observed that, for personal, social, or cultural reasons, patients sometimes preferred to disclose critical information to nonphysician members of the team.

I think that a patient doesn’t know how to tell a doctor or clinical staff the bottom line. . . . The doctor is who you’re supposed to tell all the stuff to, but what we hear all the time is “I never told the doctor, but can you tell them?” (MA, Site 7, High function and high culture)

When staff felt *empowered* to speak up to their teammates they could use this uniquely held knowledge to contribute to a patient’s care. For example, a Spanish-speaking patient repeatedly missed appointments for a diagnostic colonoscopy. The front desk staff member on her team, also a native Spanish speaker, eventually discovered that no one in the endoscopy department spoke Spanish and took it on herself to navigate her through the procedure successfully.

Practices’ use of data for continuous improvement was a particularly vivid illustration of the mutually reinforcing relationship between functional and cultural change. For high-performing practices, information sharing provided an opportunity to strengthen both technical and relational skills. Data became a tool that enabled individuals and teams to make sense of their work and practice environment, visualizing successes, and locating barriers.

We have become data fanatics. There’s constantly some data being collected; some data being analyzed. (RN, Site 12, High function and high culture)

Lower performing practices failed to establish an information sharing feedback loop. In contexts where functional change processes were neglected, practices often found that patchy data collection was a stumbling block in their aspirations for change. Without consistent feedback, projects could end up in a perpetual cycle of starting or restarting. More perniciously, when cultural change processes were overlooked, practices were more likely to view data with suspicion—it was perceived as a threat rather than helpful feedback. Practice sites that relied heavily on the mutual support of cultural and functional change in capacity building were able to tolerate uncertainty and the risk of failure when they felt they had the tools (PDSA, meetings) to work through challenges.

Team formation and continuous improvement efforts built on each other to help practices achieve effective team-based care. Within these two domains, functional and cultural change processes interacted in distinct ways, but they also interacted *across* domains. Cultural change processes played a more prominent role in team formation; then, as teams began to build improvement capacity, functional changes became more significant. The largely functional changes required for teams to become an engine for improvement around quality and efficiency depended on the cultural base of team formation.

### Taxonomy of Approaches to Change: Four Ways to Approach Teaming

Practices varied in the extent to which they had pursued cultural and functional change, clustering in three groups that suggested a taxonomy of practice approaches to change. One approach prioritized both cultural and functional change. Two other approaches were characterized by asymmetric change: practices prioritizing cultural change over functional change, and practices prioritizing functional change over cultural change. A fourth approach would be practices undertaking neither type of change, although we did not observe this fourth type in our sample, not surprisingly because all AIC participants had committed to attempting transformation.

**Figure 1.**
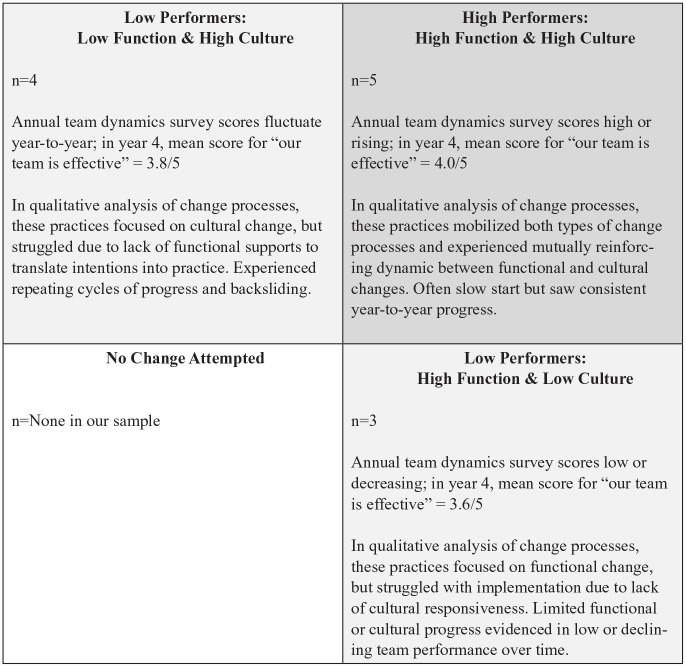
Taxonomy of practice approaches to change. *Note. n* = number of practices in study sample (out of *n* = 12 practices in total) with each combination of change characteristics.

We use this taxonomy of approaches to change to summarize survey data on team performance and qualitative data evidencing characteristics associated with effective team-based care (patient-centered care and staff satisfaction) and capacity for continuous improvement (Figure 1). High performers on the team dynamics survey fell in the “high culture, high function” quadrant of our change taxonomy and exhibited strongest qualitative evidence of effective team-based care. These were the sites that mobilized cultural and functional changes in a virtuous cycle. For example, making functional changes around role revision (MA identified as team member and role includes generating improvement ideas and actions), cultural changes related to shared authority (independent initiative by MA and front office staff welcomed), functional change around continuous improvement skills (all staff know how to do a PDSA) and a cultural openness to experimentation (“we can just do it”).

If I want to do a PDSA, I just say, “I’m doing a PDSA.” And if the MA on my team wants to do one, or the front office person comes up with a good idea, I’d say, “oh, that’s a great PDSA.” We can just do it—you don’t have to have a meeting to have an idea. (Physician, Site 7)

The two asymmetric approaches to change correlated with lower performance in the team dynamics survey, but exhibited distinct patterns of scoring that corresponded with the two change types. The group with fluctuating team dynamics scores but good professional satisfaction consisted of four practices that prioritized cultural change over functional change. The group with low team dynamics scores and weak professional satisfaction comprised the practices that prioritized functional change over cultural change. Qualitative data from these two groups of practices indicated that they did not consistently evidence traits of effective team-based care; they did not build a sound enough base in team formation to support growth in continuous improvement capacity.

## Discussion

This study investigated the processes by which 12 primary care practices undertook the transition to team-based care and the impact of their approaches on the effectiveness of the resulting teams. Specifically, we aimed to better understand why team-based primary care transformation initiatives produce variable results despite fairly detailed knowledge of the elements of effective primary care teams. Our analysis suggests that variation was largely driven by how a practice deployed functional and cultural change processes. Prioritization of both change types in team formation and improvement capacity building engendered a virtuous cycle: both were necessary ingredients for successfully creating effective team-based care.

AIC practices followed an evidence-based blueprint for team formation, including teamlets (Rodriguez et al., 2015); teamlet huddles at the beginning of a clinic session (Gale et al., 2015; Rodriguez et al., 2015); structured team meetings focused on QI (Grace et al., 2014, 2015; Helfrich et al., 2016); protected time for practice-level and organizational leaders to commit to leading change (Grace et al., 2014; Stockdale et al., 2018); and, finally, the learning collaborative itself (Stockdale et al., 2018). While teams were aware of and tried to incorporate best practices, this was not sufficient for achieving effective team-based care. Despite receiving the same intervention, we found considerable heterogeneity across sites that did not align with structural characteristics like practice size or patient population, or other non-AIC QI experience. Instead, practices’ approach to transformation activities, and specifically the ways in which they mobilized functional and cultural change processes, drove the effectiveness of the resulting teams. In short, it matters not only *what* practices change, but *how*. By focusing on change processes, we are able to add greater specification to practical aspects of transformation, as well as illustrate the interlocking relationships between changes that characterize complex adaptive systems.

For example, one desirable feature of primary care transformation is use of data to drive improvement (Gale et al., 2015). Our analysis unpacks the multiple distinct and interactive changes underpinning a team’s ability to use data. Functional changes include access to data across team members, assignment of data collection responsibilities, and time to discuss and respond to the data as a team. To use data constructively, functional changes need to be supported by cultural changes that enable data to be understood as a tool facilitating experimentation and learning, sometimes through failure. While the specifics of the change processes elaborated in this article are most relevant to primary care teams, the more general recursive dynamic between functional and cultural change is relevant to the formation of high-performing teams in a range of settings.

### Implications for Research

Despite wide agreement on ingredients necessary to create primary care teams, in practice, consistently implementing effective team-based care remains elusive (Dale et al., 2016; Friedberg et al., 2014; Friedberg et al., 2015; Grace, Rich, Chin, Rodriguez, 2016; Helfrich et al., 2014; Jaen et al., 2010). Drawing on complexity theory, we postulated that primary care team formation is a complex change process shaped not only by component parts but also by interactions among them (Davidoff, 2009; Pisek & Greenhalgh, 2001; Stroebel et al., 2005). Our findings contribute to theory on complex change by showing how functional and cultural change processes interact to generate distinctive transformation results in the context of primary care team formation. Future research can draw on the taxonomy of approaches to change (high functional and cultural change, high-functional change but low-cultural change, low-functional change but high-cultural change, and no change) to inform and evaluate transformation efforts.

Though our research found heterogeneity in functional and cultural change processes was key to explaining differences in the effectiveness of primary care practices’ efforts to deliver team-based care, we acknowledge that these distinctions do not explain all variation. While deemed out of scope for our discussion in this article, contextual factors clearly shaped transformation efforts. All participating practices were in the beginning stages of transformation to medical home, team-based care type models. However, there were other contextual factors that varied across sites with implications for how practices could undertake change. For example, practice sites’ health systems often specified scope of practice policies that circumscribed role responsibilities, limiting the possibilities for delegation across roles. Health systems also controlled data systems and referral processes, constraining the ability of practice sites to develop continuous improvement capabilities. Similarly, efforts to create shared accountability within teams were limited by prevailing fee-for-service payment systems, which continue to use physician payment as the principal lever for controlling clinical practice. Future research could explore the starting conditions that predispose practices sites to emphasize function or culture, or that pose particular obstacles for deploying one or the other (Ovretveit, 2011).

### Implications for Practice

While the prospect of simultaneously initiating both functional and cultural change may be daunting, our results suggest that committing to both pays off. Practices undertaking team-based care may find it useful to think of team formation as a change within a complex adaptive system rather than a set of discrete interventions. Our detailed findings further provide a roadmap for practices seeking to offer effective team-based care. Consistent with prior literature, our findings suggest that practices forming teams should pursue functional change characterized by delegation, shared access to data (electronic health records), and team time (Helfrich et al., 2014; Helfrich et al., 2016). Concurrent cultural change should include shared authority, staff engagement, and physician leadership. Commitment to patients is an essential point of common ground and trust building. Practices should also seek to build improvement capacity, including functional change through developing continuous QI skills, data collection, and formalized meeting structures, and cultural transformation through openness to experimentation, willingness to fail, and receptivity to data as a tool rather than a punishment.

### Implications for Policy

Within a structured learning collaborative where all participants received the same guidance and support, and—on paper at least—had organized providers and staff into teams, we found significant heterogeneity in the implementation of a primary care transformation program and the quality of resulting teams. This suggests there may be similar diversity—and efficacy—among practices attesting to team-based care in credentialing programs like PCMH. We may thus be counting “false positives” by using medical home accreditation as a proxy measure for team-based care. Assessing transformation may require a more nuanced analysis of practice change than is captured in accreditation documentation.

Furthermore, we found that successful practices showed a change pattern of starting slowly before building momentum. Large-scale evaluations of PCMH-type models that take place after the first year or second of an intervention may be too early to detect success or failure. Three years into the AIC, even the most advanced practices in our sample reported feeling like they were early in their transformation. Policies seeking to assess successful transformation to effective team-based care should be sensitive to heterogeneity in implementation of team-based care, and the time interval which may be required to capture progress.

### Limitations

First, our study sample drew from academically affiliated primary care practices participating in a learning collaborative, which included financial support and technical assistance for participants that may not available to all primary care practices (although the level of support was consistent with similar transformation endeavors, such as payer-led initiatives). Having protected time for leaders to devote to transformation is a prominent enabler of team formation, reported in our own analysis and in other research (Stockdale et al., 2018). The advantages of protected time could be difficult for other organizations to replicate without a grant or other resources. Likewise, the technical assistance provided through the AIC gave participants access to useful expertise that might not be available in other settings. The academic nature of the practices meant that many employees, especially physicians, worked part time. This meant there was often limited continuity in the people working together on a given team from day to day. Second, our interview sample included a large proportion of physicians. Physicians, due to their status, were less dependent on teams than other staff members and, as a group, were the most circumspect in their endorsement of the transition to team-based care. Our emphasis on physician perspectives may thus understate the impact of team formation on practice transformation. Future work would benefit from greater attention to nonphysician personnel. Third, we relied on self-reported data in both the surveys and interviews, though we did confirm performance designations with improvement coaches who observed teams’ transformation efforts over time; direct observation to assess whether individuals’ perceptions of team quality align with observed behavior might be useful. Despite these limitations, this study offers valuable insight into the processes underpinning the transition to effective teams in primary care, an important organizational and policy priority.

## Conclusion

Forming effective teams is a central priority of primary care transformation, but practices often struggle to achieve effective team-based primary care. We showed that transitioning to effective team-based care depends on deploying mutually reinforcing functional and cultural change processes to achieve team formation and build capacity for improvement. Greater attention to how practices deploy cultural and functional change processes, and in what combination, could offer corrective insights to support practice transformation, and help deliver on the promise of team-based care for patients.

## Supplemental Material

Appendix_change_processes_enabling_team-based_care_revised_vSUBMIT – Supplemental material for A Mixed Methods Study of Change Processes Enabling Effective Transition to Team-Based Care Supplemental material, Appendix_change_processes_enabling_team-based_care_revised_vSUBMIT for A Mixed Methods Study of Change Processes Enabling Effective Transition to Team-Based Care by Michael Anne Kyle, Emma-Louise Aveling and Sara Singer in Medical Care Research and Review
