# Supplementary material for: A Mixed Methods Study of Change Processes Enabling Effective Transition to Team-Based Care
Source: Med Care Res Rev. 2019 Oct 15;78(4):326–37. doi: 10.1177/1077558719881854 (PMC8295944; doi:10.1177/1077558719881854)
Supplement: Appendix_change_processes_enabling_team-based_care_revised_vSUBMIT – Supplemental material for A Mixed Methods Study of Change Processes Enabling Effective Transition to Team-Based Care [file Appendix_change_processes_enabling_team-based_care_revised_vSUBMIT.pdf]

Appendix A. Codebook

**A priori themes**	**Emergent themes**
• Perception of transformation/team based care<br>• Team performance<br>○ Roles<br>○ Communication<br>○ Barriers<br>○ Facilitators<br>• Patient centered care<br>○ Barriers<br>○ facilitators<br>• Role of AIC<br>○ Activities associated with AIC (e.g. meetings)<br>○ QI projects associated with AIC (e.g., CRC screening)<br>○ Perceptions of AIC<br>○ Barriers<br>○ Facilitators<br>• Quality improvement<br>○ Barriers<br>○ Facilitators	• Team formation<br>○ Function<br>▪ Role revision<br>▪ Team time<br>▪ Shared access to clinical data<br>○ Culture<br>▪ Redistributing authority<br>▪ Flattening hierarchy<br>• Continuous improvement capacity<br>○ Function<br>▪ Developing continuous QI skills<br>▪ Data collection<br>▪ Practice wide meeting structures<br>○ Culture<br>▪ Openness to experimentation<br>▪ Willingess to fail<br>▪ Receptive to data<br>• Recursivity (bridges between cultural and functional codes e.g., description of data collection + receptivity to data)

Appendix B. Team Dynamics Survey Scores

	High performers (high or rising scores)				Low performers (type 1: fluctuating scores)				Low performers (type 2: low or declining scores)			
Year of Survey	**Y1**	**Y2**	**Y3**	**Y4**	**Y1**	**Y2**	**Y3**	**Y4**	**Y1**	**Y2**	**Y3**	**Y4**
N	290	299	301	304	237	242	252	229	256	210	225	177
Our team is effective, by practice	3.77	4.16	4.27	4.32	3.02	4.14	4.18	3.75	3.22	3.24	3.33	3.45
	3.53	3.91	4.03	4	3.26	3.58	3.8	3.7	3.45	3.79	3.59	3.73
	3.57	3.88	3.84	4	3.45	3.72	3.77	3.82	3.34	3.57	3.85	3.61
	3.3	3.91	3.93	3.95	3.87	3.46	3.84	3.57				
	3.12	3.67	3.76	3.91								
Teams have right skill blend & stability, by practice	3.46	3.96	4.19	4.13	3.11	4.13	4.15	3.68	3.24	3.21	3.22	3.35
	3.42	3.64	3.87	3.91	3.39	3.75	3.67	3.69	3.42	3.65	3.46	3.54
	3.29	3.66	3.77	3.88	3.23	3.66	3.81	3.71	3.4	3.5	3.64	3.38
	3.54	3.66	3.68	3.77	3.72	3.52	3.69	3.58				
	3.69	3.71	3.78	3.88								
Teams share goals and understand one another's roles, by practice	3.77	4.16	4.27	4.32	3.86	3.74	3.83	3.84	3.49	3.49	3.56	3.67
	3.68	3.87	4	3.99	3.42	3.68	3.92	3.89	3.58	3.77	3.72	3.75
	3.76	4.17	4.37	4.31	3.68	3.91	3.94	3.99	3.69	3.52	4.01	3.81
	3.76	3.84	3.86	4.1	3.27	4.17	4.44	4.04				
	3.71	3.92	4.03	4.1								
Team members communicate, resolve conflict, and hold one another accountable, by practice	3.32	3.45	3.42	3.64	2.9	3.33	3.76	3.43	3.12	3.08	3.28	3.36
	3.28	3.7	3.76	3.75	3.64	3.37	3.71	3.61	3.23	3.44	3.76	3.42
	3.29	3.64	3.71	3.82	2.76	3.96	4.18	3.7	3.4	3.61	3.39	3.67
	3.51	3.85	4.13	3.86	3.24	3.56	3.69	3.75				
	3.28	3.66	3.72	3.8								
Team members show respect and trust for one another's work, by practice	3.94	4.24	4.49	4.47	3.93	3.7	4	3.79	3.69	3.64	3.8	3.86
	3.96	4.16	4.25	4.27	3.71	3.94	4.08	4.07	3.88	3.84	4.23	3.9
	3.72	4.07	4.16	4.16	3.92	4.11	4.14	4.1	3.78	3.94	3.86	3.95
	3.79	3.97	4.06	4.15	3.7	4.37	4.39	4.16				
	3.68	3.91	4.01	4.11								

Red denotes that the practice scored in the bottom three

Appendix C. Qualitative analysis: Example quotes for team formation themes

Theme	High Function, High Culture	Low Function, High Culture	High Function, Low Culture
Role revision	The responsibility is on everyone. My nurses focus on hospital discharges and depression and abnormal PAPs. For the medical assistants, it's the colorectal screening and the mammos … And we'll have the front desk staff making well child visits. *(Nurse Manager, Site 13)*	While we are all very focused on team based care, and I think our team meetings are very active and people from all roles suggest ideas and changes, and that part is good. Distributing the work hasn't really happened as much as we would expect. *(Medical Director, Site 2)*	I think culturally many providers still feel like they own the patients and that all their staff members are supporting them. Versus all the staff members feeling like we collectively own the patients. *(Program Manager, Site 6)*
Sharing authority	We spent a solid amount of time talking about expectations around team norms in the practice. And we did a couple of workshop-type lunch meetings where we had everybody together and came up with a values statement of how we expected to handle things, and we did that around 4 specific [values]: responsibility, accountability, conflict resolution, and communication. *(Medical Director, Site 12)*	The other [thing I've learned from] the AIC is that I would never presume to tell them what the best flow chart is going be for the work. …I'm like, whoa, wait a minute – we can't even have this conversation [without] people representing all the teams that are going work on this [improvement process]. *(Medical Director, Site 8)*	Generally, what we've done is identify priorities at the practice leadership level that we'd like to do over the next twelve months and set goals and targets for ourselves on a quarterly basis and then look at how are we making progress in these ten key areas, for instance. *(Medical Director, Site 6)*
Team time	We do group huddles here, which really helps with the medical assistants' understanding what the patients are coming in for…. The MAs know what they're doing ahead of time. [Before,] you'd go	I think we've sort of lost direction – what the staff meetings are supposed to be, where they are going. I think we've had the team meetings twice in the past six months. I think the people in	When I started there were regular nursing meetings once a month, and all-staff meetings once a month. [Then, practice leaders] changed it to four meetings a month. But we never

	in and it would just be doing the vitals to remind the patient and then the physician would come out, tell them what they needed, instead this is more structured and it flows better for the MA and for the physician. *(MA, Site 7)*	charge very much acknowledge this, that they've lost their way a little bit, and they're not sure how to use the team time. *(Social Worker, Site 5)*	accomplished anything in those meetings. No one ever knew the purpose of the meeting, what we were supposed to discuss. So we spent a lot of time trying to figure out what we were supposed to be doing in this meeting. *(RN, Site 10)*
Staff engagement	We said don't worry about your quality numbers, do whatever you want, do whatever you think is going to make your team function better. We are still going to watch your numbers, of course. But what we saw is that teams who did that had more engaged team members, and their numbers actually went up. *(Medical Director, Site 13)*	I'm concerned that it's not engaging the other team members as much. I have to be the most engaged person as the provider, and I haven't had really much opportunity to ask the other team members what they think about this, but I mentioned to our medical director that this is something I want to be careful of; that it doesn't suddenly feel like the providers have complete ownership of the patient. *(Physician, Site 2)*	There are certainly staff members that are very engaged with patients. And then there's staff members that collect a paycheck. *(Program Manager, Site 6)*
Physician leadership	We've tried to make it pretty clear to people [what is] not acceptable behavior, pulling people aside...that's the way that actual accountability happens. It has to come from leadership. I don't think it works to put that responsibility on the staff, because there is such a hierarchy in medicine, it has to be someone	I don't lead by making orders. That just doesn't work. It doesn't work with a bunch of academics who have feelings about the way things should be done, and academic freedom, and professional freedom, and all that stuff. I am very careful when I tell people "You have to do X, Y, or Z." I don't do that very often. When I do it, they still get mad at	There's certainly different expectations about how front line staff are brought in line, and how providers are brought in line… There's so much tiptoeing around, trying to approach them in such a way that doesn't ruffle any feathers. *(Program Manager, Site 6)*

	who actually has some authority. *(Medical Director, Site 12)*	me, but they'll do it. *(Medical Director, Site 5)*	
Access to clinical data	[The MA] will circle what he thinks are the appropriate vaccines based on his review of the chart using our standard immunization schedule, he is pretty savvy about catching people up when he realizes they haven't gotten a vaccine. (*Medical Director, Site 12)*	We don't rely completely on the technology, but the doctor will put the Huddle Note in. One of my doctors always runs behind. He'll put a note in saying, "If I'm running behind, can you do this, this, and this for this patient? …It's really great. *(LPN, Site 5)*	You followed your provider's patients. If your provider had 20 patients, you know those 20 patients belong to you. You have to make sure that the health maintenance and the problem list, as well as that smart steps, are all documented clearly, so everyone can see it. *(RN, Site 10)*

Appendix D. Qualitative analysis: Example quotes for building team capacity for improvement themes

Theme	High Function, High Culture	Low Function, High Culture	High Function, Low Culture
Improvement skills	I was the biggest opponent. I hated PDSAs. I'm a do-er, let's just do it. Now I'm like, "Why do it if we don't know what is making progress?" So I tell my Medical Director that I'm one of the people who drank the Kool-Aid. PDSA, it works. *(Nurse Manager, Site 13)*	One of the things we've talked about that we want to do is quality improvement projects [like] diabetic foot exams. That's something that would be in the wheelhouse of a medical assistant – it would be kind of fun, very useful for them, great for me for it to be done. For multiple reasons, it isn't feasible in the practice setting we are in– it's considered an assessment that's beyond the skill set of a medical assistant, which I disagree with. *(Physician, Site 5)*	The trainings seem to be like a little bit random—it's more like things that you have to do from a not quality improvement standpoint but the quality control side of things. *(Physician Assistant, Site 10)*
Meeting structures	But those [care teams] are the little siloes. Now there's this little	That's where I think the fits and starts with the staff meeting comes	I think we are still trying to figure out what's the best way to use

	direct line [the innovation team] between them all, connecting them all, which is a pretty neat thing. *(RN, site 4)*	in. We sort of have these projects and a lot of energy goes into them, and then they get dropped. I kind of feel like complex care got dropped in the middle. (Social Worker, Site 5)	those meeting times and how it could actually contribute to improved quality. *(Social Worker, Site 10)*
Openness to Experimentation	If I want to do a PDSA, I just say, "I'm doing a PDSA." And if the MA on my team wants to do one, or the front office person comes up with a good idea, I'd say, "oh, that's a great PDSA." We can just do it—you don't have to have a meeting to have an idea. *(Physician, Site 7)*	We do have a culture where our people will say, "Try it." That's kind of a cool new phrase, "Let's try it." *(Physician, Site 8)*	Another barrier is that the QI team can be a little intimidating, and I've learned this through conversations with other individuals within the clinic. I think some people see it as very high level when, in actuality, this is like, "Look, just give us your ideas, your thoughts..." And a lot of people think they really can't contribute anything. *(Project Manager, Site 3)*
Willingness to fail	We had to depersonalize it….So we gave a sheet a paper to each of the teams, [to write] the problems, list the solutions and … find an agreement. We had four mini groups working at the same time to figure out what they wanted to do. *(RN, Site 12)*	We do a tally [of PDSA ideas] and [choose what to try]. And check to see if it is working. If it is working, we take it. If it doesn't work, we try to find something else. *(Administrative Assistant, Site 1)*	[Quote not available in these practices' data]
Data collection capacity	I think the CRC process has been great because we've looked at things like how we communicate with patients. How do we engage providers in putting things into structure fields? How do we put outreach mechanisms in place?	But it's not systematic. So I think that what we are having trouble with is, we don't have a base of information that's big enough to start looking at parameters, or to start looking at risk and disease. *(Physician, Site 1)*	And trying to look at how we screen patients that don't already have a diagnosis of depression. And I have no data on how many—what percent. Outside of like very specific claims data that I don't know if we have any

	And then it gets into billing, and how data moves through the system. *(Care Manager, Site 7)*		confidence in, or limited confidence in, I have no data on it. *(Program Manager, Site 6)*
Data as a valued tool	We have become data fanatics. There's constantly some data being collected, some data being analyzed. We have used the knowledge that we gained through the AIC and through those many team meetings to request that our new EHR have capabilities that before we wouldn't have thought of [before]…we have to be able to run reports. We have to be able to collect data." *(RN, Site 12)*	I think that using data to refine your efforts, so looking at the data to say we have 0 care plans done. That's our data point right now. So what we are doing right now isn't working, let's try something next. *(Physician, Site 2)*	We have done limited presentation of data at the provider level. Say you have two MAs, and you say this MA's at 80%, this MA's at 60%. The MA's response is, "How do I get to 80%?" If you say that to two providers, both providers will say, "Where did this data come from? How do I know this is the right data?" *(Program Manager, Site 6)*
